# Drug Seeking and Relapse: New Evidence of a Role for Orexin and Dynorphin Co-transmission in the Paraventricular Nucleus of the Thalamus

**DOI:** 10.3389/fneur.2018.00720

**Published:** 2018-08-28

**Authors:** Alessandra Matzeu, Rémi Martin-Fardon

**Affiliations:** Department of Neuroscience, The Scripps Research Institute, La Jolla, CA, United States

**Keywords:** paraventricular nucleus of the thalamus, orexin, dynorphin, drug addiction, drug-seeking behavior, natural reward

## Abstract

The long-lasting vulnerability to relapse remains the main challenge for the successful treatment of drug addiction. Neural systems that are involved in processing natural rewards and drugs of abuse overlap. However, neuroplasticity that is caused by drug exposure may be responsible for maladaptive, compulsive, and addictive behavior. The orexin (Orx) system participates in regulating numerous physiological processes, including energy metabolism, arousal, and feeding, and is recruited by drugs of abuse. The Orx system is differentially recruited by drugs and natural rewards. Specifically, we found that the Orx system is more engaged by drugs than by non-drugs, such as sweetened condensed milk (SCM) or a glucose saccharin solution (GSS), in an operant model of reward seeking. Although stimuli (S^+^) that are conditioned to cocaine (COC), ethanol, and SCM/GSS equally elicited reinstatement, Orx receptor blockade reversed conditioned reinstatement for drugs vs. non-drugs. Moreover, the hypothalamic recruitment of Orx cells was greater in rats that were tested with the COC S^+^ vs. SCM S^+^, indicating of a preferential role for the Orx system in perseverative, compulsive-like COC seeking and not behavior that is motivated by palatable food. Accumulating evidence indicates that the paraventricular nucleus of the thalamus (PVT), which receives major Orx projections, mediates drug-seeking behavior. All Orx neurons contain dynorphin (Dyn), and Orx and Dyn are co-released. In the VTA, they play opposing roles in reward and motivation. To fully understand the physiological and behavioral roles of Orx transmission in the PVT, one important consideration is that Orx neurons that project to the PVT may co-release Orx with another peptide, such as Dyn. The PVT expresses both Orx receptors and κ opioid receptors, suggesting that Orx and Dyn act in tandem when released in the PVT, in addition to the VTA. The present review discusses recent findings that suggest the maladaptive recruitment of Orx/Dyn-PVT neurotransmission by drugs of abuse vs. a highly palatable food reward.

## Introduction

Drug addiction is a chronic relapsing disorder that is characterized by persistent drug seeking and use (1,4). Relapse vulnerability is a challenge for the successful treatment of substance use disorder, and relapse prevention has emerged as a central focus of treatment and medication development efforts (4, 5). Central issues in addiction research involve clarification of the neurobiological mechanisms that underlie the chronic relapsing nature of addiction and the identification of pharmacotherapies for relapse prevention.

Progress has been made in identifying the neurocircuitry that mediates craving, drug seeking, and relapse. Human functional brain imaging studies (6–8) and rodent studies of Fos expression as a marker of neural activation (9–11) have identified interconnected cortical and limbic brain regions that are activated during drug cue-, drug priming-, and stress-induced reinstatement. This circuitry includes the medial prefrontal cortex (mPFC), basolateral amygdala (BLA), central nucleus of the amygdala (CeA), bed nucleus of the stria terminalis (BNST), ventral tegmental area (VTA), nucleus accumbens (NAC), hippocampus (HIP), dorsal striatum (DS), hypothalamus (HYP), and thalamus [THAL; (12–25)].

**Figure 1:**
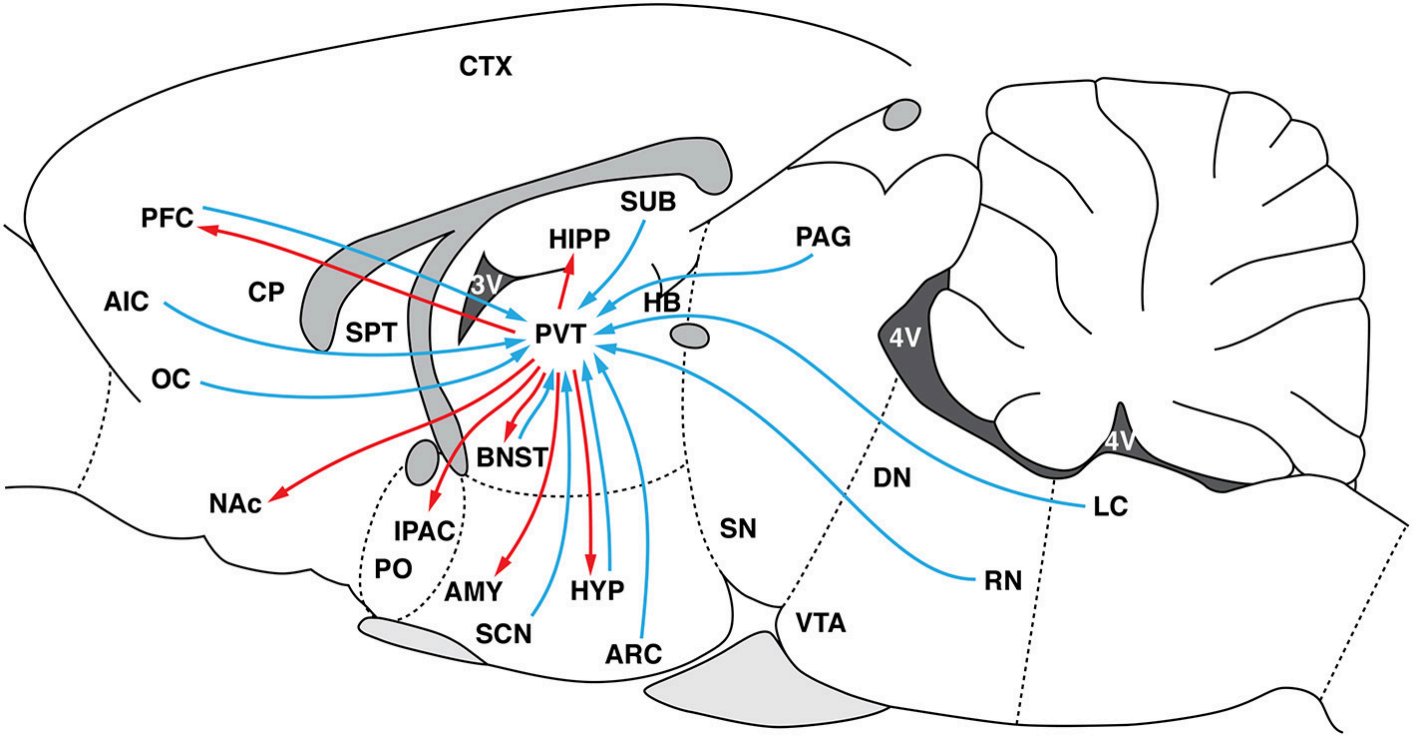
Schematic representation of major PVT efferents (red arrows) and afferents (blue arrows). HYP, hypothalamus; PVT, paraventricular nucleus of the thalamus; ARC, arcuate nucleus of the hypothalamus; LC, locus coeruleus; RN, raphe nucleus; PN, parabrachial nucleus; CTX, cortex; PFC, prefrontal cortex; AIC, agranular insular cortex; OC, orbital cortex; CP, caudate-putamen; SPT, septum; AMY, amygdala; PAG, periaqueductal gray; BNST, bed nucleus of the stria terminalis; NAc, nucleus accumbens; IPAC, interstitial nucleus of the posterior limb of the anterior commissure; PO, preoptic area; HIPP, hippocampus; SUB, subiculum; SN, substantia nigra; VTA, ventral tegmental area, SCN, suprachiasmatic nucleus.

The paraventricular nucleus of the thalamus (PVT) is a strategic interconnected neuroanatomical region among thalamic nuclei (Figure 1) that influences other structures that have been implicated in drug-seeking behavior (26–28). Of particular interest, the PVT receives abundant innervation by hypothalamic orexin (Orx) neurons (29). Orexin system function has been positively related to states of arousal and maintaining the waking phase (30). Orexin has also been reported to modulate reward function, particularly drug-seeking behavior [for review, see (31)]. Orexin neurons express dynorphin [Dyn; (32)]. In contrast to Orx, Dyn promotes depressive-like behavior and is involved in mediating the aversive effects of stress (33). The PVT expresses Orx receptors (OrxRs) and κ opioid receptors (KORs), suggesting that Orx and Dyn have a functional interaction in the PVT.

Neural systems that are involved in processing natural rewards and drugs of abuse overlap. Neuroplasticity that is caused by drug exposure may be responsible for maladaptive, compulsive, and addictive behavior (34–37). One important consideration when studying the brain mechanisms that control dug-seeking behavior is to differentiate the mediation of drug-directed behavior from “normal” appetitive behavior. Drugs neuroadaptively influence neural systems that regulate motivation that is normally directed toward natural rewards. Neuroplasticity of this circuitry may be responsible for the maladaptive compulsive behavior that characterizes addiction. This review summarizes recent findings that suggest maladaptive recruitment of the PVT by drugs of abuse. More specifically, this review focuses on Orx transmission in the PVT. There is a functional interaction between Orx and Dyn that impacts drug-seeking behavior. The present review also discusses recent data that demonstrate a differential role for Orx and Dyn in behavior that is directed toward conventional reinforcers. The terms “conventional reinforcer” and “natural reward” are loosely defined in the present review as a non-drug condition (e.g., a sweet, highly palatable solution) that serves as a comparison control for the drug.

## Motivational effects of drugs of abuse implicate the orexin system

Orexin A (OrxA or hypocretin-1 [Hcrt1]) and orexin B (OrxB or hypocretin-2 [Hcrt2]) are obtained through the proteolytic cleavage of the common precursor prepro-Orx (38–40) and post-translational products of the orexin (*Orx*) neuropeptide precursor gene. The hypothalamic neuropeptides OrxA and OrxB regulate energy metabolism, arousal, and feeding (41–48). To date, orexin receptor 1 (OrxR1) and OrxR2 have been identified (44, 49, 50). OrxR1 has higher affinity for OrxA (20–30 nM) than OrxB (10- to 1,000-fold lower). OrxR2 has similar affinity for OrxA and OrxB [40 nM range; (44, 49, 50)]. Orexin cell bodies have been identified in the lateral hypothalamus (LH), perifornical hypothalamus (PFA), and dorsomedial hypothalamus (DMH). The LH is associated with reward and motivation (51), and the PFA and DMH are involved in arousal regulation and stress responses (52, 53). Orexin neurons project to the PVT, NAC shell (NACsh), ventral pallidum (VP), VTA, CeA, BNST, and mPFC (52, 54). Over a decade ago, Orx neurons were shown to play a role in modulating reward function and particularly drug-directed behavior (55). Orexin neurons in the LH are activated by stimuli that are predictive of food, morphine, cocaine (COC), and ethanol [EtOH; (19, 55–58)]. Consistent with these observations, intra-VTA microinjections of OrxA produced the renewal of morphine-induced conditioned place preference [CPP; (59, 60)]. Intra-LH administration of the OrxR1 antagonist SB334867 decreased morphine-induced CPP (55). SB334867 also blocked the acquisition of COC-induced behavioral sensitization and the COC-induced potentiation of excitatory currents in dopamine (DA) neurons in the VTA (61). Furthermore, intra-VTA SB334867 administration reduced the motivation to self-administer COC and decreased the COC-induced enhancement of DA signaling in the NAC (62). OrxR1 blockade decreased both EtOH (63) and nicotine (64) self-administration and prevented the reinstatement of COC and EtOH seeking that was induced by cues and stress (57, 63, 65–69). Overall, the Orx system has been implicated in the neurobehavioral and motivational effects of drugs of abuse (61, 70, 71). Orexin projection sites overlap with neural systems that mediate drug craving and seeking (12, 14, 17, 18, 21, 24).

## Neuronal circuitry that encodes natural rewards may be usurped by drugs of abuse

Neural systems that are involved in processing natural rewards and drugs of abuse overlap. Exposure to drugs of abuse induces neuroadaptations that can cause compulsive-like behavior (34–37). Evidence has shown that the Orx system is recruited by drugs of abuse. A recent study from our laboratory demonstrated differential recruitment of the Orx system by motivational states that were induced by discriminative stimuli that were paired with COC vs. a highly palatable food reward (19). Although weak behavioral effects of OrxR blockade were described for COC (62, 72–74) and EtOH (75, 76) intake, pharmacological manipulation of the Orx system significantly affected the motivational effects of drug-predictive stimuli in CPP and reinstatement studies (55, 57, 63, 66–69). Our laboratory utilized an operant model of reward seeking and found that drugs of abuse engage the Orx system more than non-drugs [e.g., sweetened condensed milk (SCM) and a glucose saccharin solution (GSS); (77)]. For example, although stimuli (S^+^) that were conditioned to COC, EtOH, and SCM/GSS were equally effective in eliciting reinstatement, systemic SB334867 administration selectively reversed conditioned reinstatement for drugs vs. non-drugs (66, 67). SB334867's preferential effect suggests that drugs neuroadaptively alter neurocircuitry that regulates motivation that is normally directed toward natural rewards, and such alterations can only be revealed by pharmacological manipulations. Notably, baseline levels of responding during self-administration in these studies were considerably higher with SCM than with COC, which could be interpreted as the sweet solution's not really being a “non-drug condition” but rather much more reinforcing than the drug of abuse. However, reinforcer efficacy and the rate of responding on fixed-ratio schedules are not necessarily correlated (78, 79). In *ad libitum*-fed animals, SCM at the concentration that was used herein produced breakpoints on a progressive-ratio schedule that were similar to breakpoints that were produced by COC (80–82); thus, the acute reinforcing effects of SCM and COC were comparable. Additionally, stimuli that were conditioned to this COC dose and SCM concentration produced identical conditioned reinstatement, suggesting that reliable and comparable conditioning effects under these conditions occurred for COC and SCM despite the difference in baseline levels of responding that were maintained by these reinforcers (66, 83, 84). However, when presented repeatedly, environmental stimuli that were conditioned to drugs of abuse produced perseverative, highly extinction-resistant reward seeking, whereas behavior that was controlled by stimuli that were associated with conventional reward extinguished rapidly in the absence of primary reinforcement (19, 77). This supports the conclusion that the perseveration of reinstatement or reward craving that results from reward-environment associations is a phenomenon that is preferentially linked to drugs of abuse and occurs independently from the initial primary reinforcing strength of the substance that maintains behavior during self-administration. Moreover, for COC, the percentage of Fos^+^/Orx^+^ cells was significantly elevated in the LH, DMH, and PFA, and this effect was not observed in SCM rats (19). These findings suggest a role for the Orx system in perseverative, compulsive-like drug seeking but not behavior that is motivated by non-drug palatable food.

## The paraventricular nucleus of the thalamus controls drug-seeking behavior

The PVT is part of dorsal midline thalamic nuclei and adjacent to the dorsal aspect of the third ventricle. The PVT plays a major role in regulating arousal, attention, states of awareness, food consumption, and energy balance (28, 85–88). Midline and intralaminar thalamic nuclei were previously hypothesized to participate in processing non-discriminative nociceptive inputs (89), and each nucleus has subsequently been shown to innervate functionally distinct areas of the cortex and striatum (28, 87, 90).

As shown in Figure 1, the PVT receives projections from brainstem regions that are involved in arousal and autonomic nervous system function (91–96). Through its projections to the NAC and PFC (26,27, 93, 97–104), the PVT affects cortico-striatal mechanisms that are related to reward and motivation (13, 105, 106).

The PVT receives large and distinct inputs from several areas of the HYP, including the suprachiasmatic, arcuate, dorsomedial, and ventromedial nuclei, as well as preoptic and lateral hypothalamic areas (29, 91, 93, 107–111). These structures control the expression of motivated behavior (112). The PVT is the target of hypothalamic Orx neurons, especially from the LH (29). Additionally, the PVT interfaces HYP and cortical-striatal projections that integrate energy balance, arousal, and food reward [e.g., (113)].

The PVT has been consistently shown to be activated during periods of arousal and stressful conditions (114–119). The PVT was also shown to mediate activity of the hypothalamic-pituitary-adrenal axis in response to chronic stress (114, 120, 121). The PVT was not initially thought to be part of the “neurocircuitry of addiction,” but it has been subsequently shown to play a key role in modulating drug-directed behavior. The PVT sends projections to the NAC, CeA, BNST, and PFC, brain regions that are involved in controlling drug-seeking behavior [Figure 1; (26–28)]. Previous studies reported that the PVT is selectively activated during EtOH-seeking behavior (56, 122). Our recent studies found potent and selective PVT activation during COC-seeking behavior but not SCM-seeking behavior (20). The PVT was recruited during the conditioned reinstatement of COC and SCM seeking, but its activation was correlated with COC- but not SCM-seeking behavior (20). These findings were extended by showing that transient inactivation of the PVT selectively prevented the conditioned reinstatement of COC- vs. SCM-seeking behavior (123), further demonstrating an important role for the PVT in drug-seeking behavior.

The PVT as a whole plays a role in the mediation of drug-seeking behavior. Accumulating evidence shows that Orx transmission in the PVT is specifically implicated in the control of drug-seeking behavior. There is a major Orx projection from the LH to the PVT (29, 124) and from the PFC to the PVT (Figure 1). These connections are hypothesized to modulate the expression of emotional and motivated behaviors (125). The PVT has been proposed to be a key relay that gates Orx-coded reward-related communication between the LH and both the NAC and DS (113).

Recruitment of the Orx system by drugs of abuse may induce neuroadaptations that slant its function toward drug-directed behavior. This may explain the greater sensitivity of the Orx system to OrxR antagonism for drug-seeking behavior vs. natural reward-seeking behavior (66) and could explain why transient inactivation of the PVT prevented COC conditioned reinstatement and not behavior that was motivated by stimuli that were paired with a highly palatable food (123, 126). Orexin transmission in the PVT is directly implicated in COC-seeking behavior. A recent study from our laboratory showed that Orx administration in the PVT reinstated (primed) COC-seeking behavior in animals that had a history of COC dependence (127). Surprisingly, however, Orx's priming effect was prevented by co-administration of the OrxR2 antagonist TCSOX229 and not by SB334867. Intra-PVT administration of SB334867 did not exert such effects, thus confirming the results of a previous study (128) that found that intra-PVT SB334867 administration did not impact cue-induced COC-seeking behavior, thus suggesting a key role for OrxR2 signaling in the PVT in COC-seeking behavior.

The PVT receives innervation from the suprachiasmatic nucleus [SCN; the circadian pacemaker in the mammalian brain; (129, 130)]. The studies that are cited above were performed during the rats' active (dark) phase to avoid behavioral confounds that could be caused by circadian oscillations of neuronal activity in the PVT. Importantly when designing such experiments, differences in intrinsic electrical properties of PVT neurons were described in slices that were collected at different time points. For example, when rats are active during their dark phase, neurons have a more depolarized resting membrane potential and lower resting membrane conductance [for details, see (131)]. The consequences of diurnal changes in PVT neurons and the ways in which the role of the PVT in the circadian cycle affects drug-seeking behavior should be investigated in future studies.

## Cocaine-seeking behavior in the paraventricular nucleus of the thalamus is mediated by orexin receptor 2

Our laboratory has found that intra-PVT OrxA administration primed COC-seeking behavior in rats with a history of COC dependence. Co-administration of the OrxR1 antagonist SB334867 and OrxA did not affect COC seeking, whereas the OrxR2 antagonist TCSOX229 blocked OrxA-induced COC seeking (127). The data indicate that the priming effect of OrxA administration in the PVT is mediated by OrxR2.

Intra-PVT OrxA administration primed COC-seeking behavior, implicating the Orx projection to the PVT in the control of both drug craving and relapse. Orexin neurons in the LH have been suggested to modulate ventral striatum activation via a relay through the PVT (113, 124, 132). In fact, earlier studies showed that the PVT is substantially innervated by Orx fibers that originate in the LH and PFA, and the densest innervation is found in the posterior PVT (29). Orexin neurons in both the LH and PVT were shown to be activated by EtOH-related stimuli (56). These findings suggest that Orx projections from the LH to the PVT are associated with drug-seeking behavior. Furthermore, anatomical studies showed that Orx fibers were juxtaposed with PVT-activated neurons (56). OrxA may induce its priming effects by intensifying states of arousal in rats. Orexin controls general arousal (133), and the anticipation of food reward activates OrxR-expressing neurons in the PVT in rats (134). Most neurons in the PVT are sensitive to OrxA and OrxB. One important target of Orx-activated PVT neurons is the PFC (135, 136). Cortical activation that is linked to general arousal may be facilitated by Orx inputs to the PVT (137). This may explain the reinstatement of COC-seeking behavior. Intra-PVT OrxA administration was shown to increase DA levels in the NAC (138), suggesting that the PVT may be a key relay for the effects of Orx on the mesolimbic DA system and reward-seeking behavior.

Another possibility could be that OrxA administration in the PVT induces COC-seeking behavior by increasing anxiety- and stress-like behavior. In humans, anxiety, and stress are known to induce intense craving and trigger relapse during abstinence. The PVT sends projections to the BNST and CeA that contain neurons that densely express Dyn and corticotropin-releasing factor [CRF; (100)]. Dynorphin and CRF are implicated in manifestation of the stress response and negative affective states (139–142). Orexin neurons are activated by stress exposure that is reflected by an increase in Fos expression (143, 144), suggesting that Orx transmission in the PVT is required in both behavioral and physiological responses to stressful situations. Supporting this hypothesis, OrxA and OrxB administration in the PVT produces anxiety-like behavior in rats in the open field (145) and elevated plus maze (146). These findings suggest that Orx transmission in the PVT can act as a stressor under certain conditions and thus induce COC-seeking behavior. The fact that OxR1 blockade did not prevent OrxA prime-induced reinstatement is difficult to reconcile when considering numerous studies that found that peripheral SB334867 administration blocked drug-seeking behavior (66, 68, 147, 148) and the significant expression of OrxR1 in the PVT (149, 150). Importantly, however, the lack of an effect of intra-PVT SB334867 administration confirmed earlier studies that reported similar outcomes with regard to the conditioned reinstatement of COC-seeking behavior (128), suggesting a prominent role for PVT OrxR2 signaling and not OrxR1 signaling during COC-seeking behavior.

Notably, studies of drug addiction have primarily focused on the role of OrxR1; fewer studies have examined OrxR2. OrxR2 expression, similar to OrxR1 expression, is high in the PVT (149, 150). OrxR2 antagonism decreases drug intake and drug seeking when OrxR2 antagonists are administered peripherally. Peripheral OrxR2 antagonist administration decreased EtOH self-administration, decreased the acquisition of EtOH-induced CPP, decreased the expression and reinstatement of EtOH-induced CPP (75), decreased the cue-induced reinstatement of nicotine seeking (151), and decreased heroin self-administration in rats that had 12-h access to heroin per day (152). Furthermore, intra-PVT TCSOX229 administration prevented anxiety-like behavior that was induced by footshock (146). Overall, these findings suggest that PVT OrxR2 signaling is involved in mediating both stress- and anxiety-related behavior.

## Dynorphin and orexin are co-localized and co-released and have opposite effects

**Figure 2:**
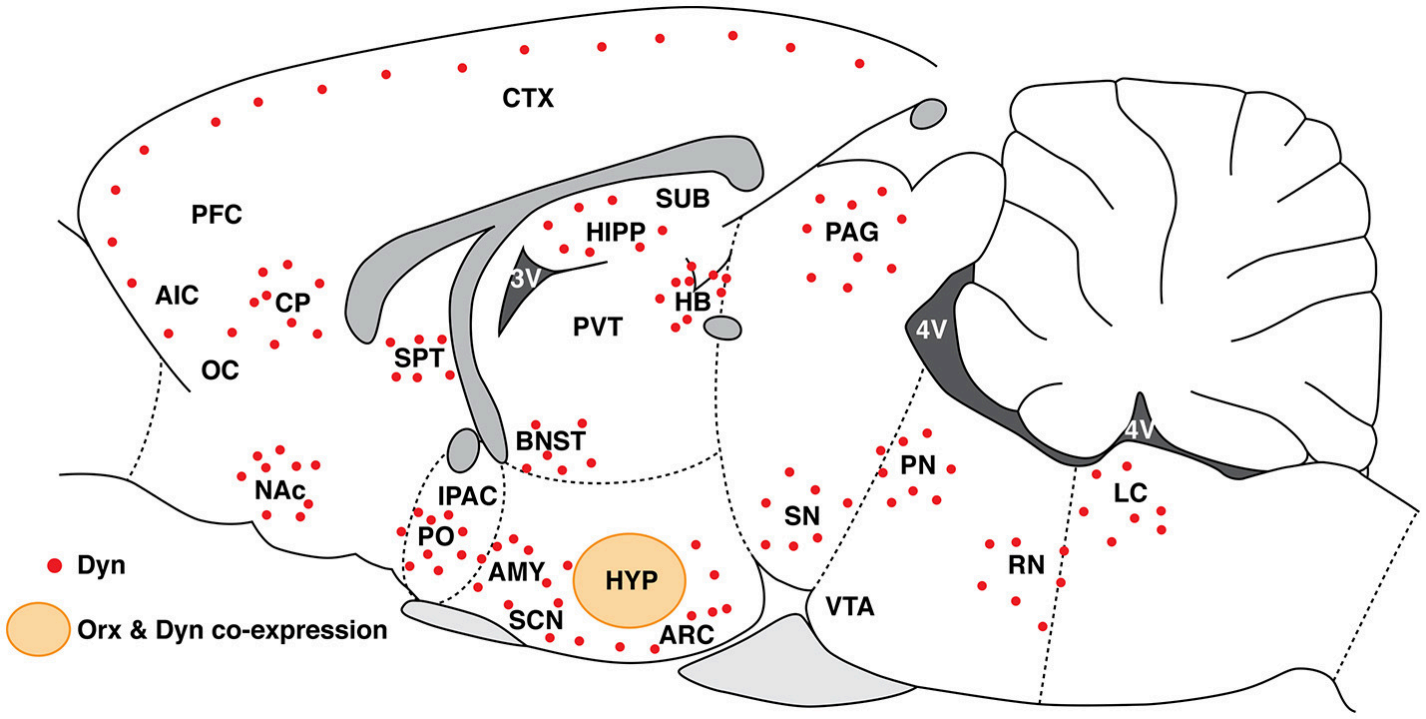
Schematic representation of Dyn distribution (red dots) along the rostro-caudal axis in brain regions that are involved in drug addiction. The orange circle represents the Orx and Dyn overlapping region in the HYP. HYP, hypothalamus; PVT, paraventricular nucleus of the thalamus; ARC, arcuate nucleus of the hypothalamus; LC, locus coeruleus; RN, raphe nucleus; PN, parabrachial nucleus; CTX, cortex; PFC, prefrontal cortex; AIC, agranular insular cortex; OC, orbital cortex; CP, caudate-putamen; SPT, septum; AMY, amygdala; PAG, periaqueductal gray; BNST, bed nucleus of the stria terminalis; NAc, nucleus accumbens; IPAC, interstitial nucleus of the posterior limb of the anterior commissure; PO, preoptic area; HIPP, hippocampus; SUB, subiculum; SN, substantia nigra; VTA, ventral tegmental area; SCN, suprachiasmatic nucleus.

**Figure 3:**
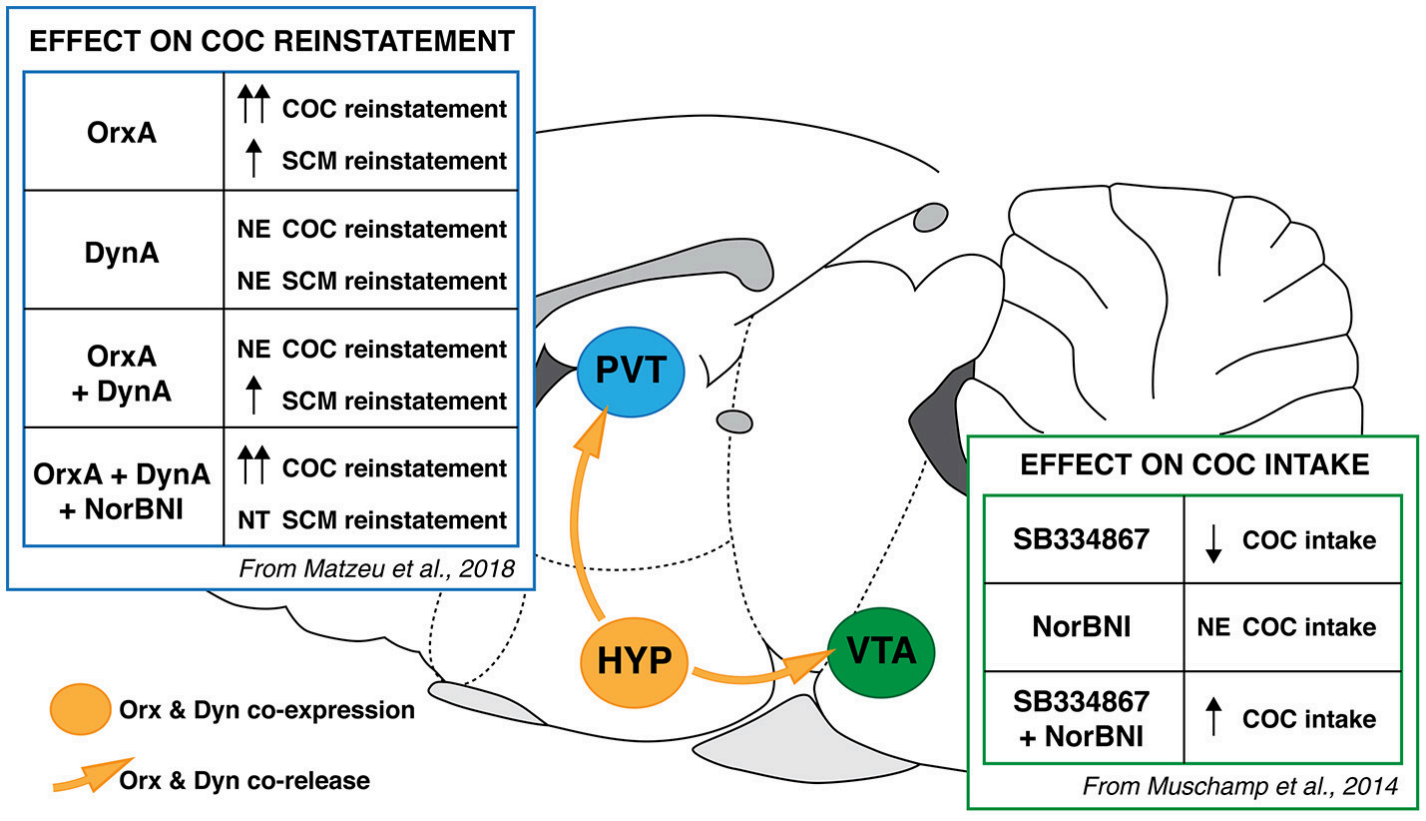
Schematic summary of Orx and Dyn interaction in COC-directed behaviors in the PVT and VTA. (PVT) In rats, OrxA injection in the PVT reinstated COC- and SCM-seeking behavior, with a greater effect in COC animals. DynA blocked OrxA-induced COC seeking but not SCM seeking. nor-Binaltorphimine (nor-BNI) did not induce or potentiate COC-seeking behavior that was induced by OrxA but reversed the effect of DynA (183). (VTA) Intra-VTA administration of SB334867 reduced COC self-administration in rats. nor-BNI administration alone had no effect but reversed the effect of SB334867 (181). PVT, paraventricular nucleus of the thalamus; VTA, ventral tegmental area; NE, no effect; NT, not tested.

The Dyn/KOR system is widely distributed in the central nervous system (153, 154). Dynorphin has received increasingly more consideration regarding its regulatory action in many functional pathways of the brain. Consistent with its localization in the hippocampus, hypothalamus, amygdala, striatum, and cortex [Figure 2; (153)], these functions are associated with learning and memory, emotional states, reward mechanisms, stress responses, and pain [for review, see (155)]. Indeed, Dyn is involved in several mood- and motivation-related pathophysiological and physiological processes (155–158). The Dyn/KOR system has been hypothesized to be a possible therapeutic target for treating neuropsychiatric disorders, including drug addiction (157–161). Dynorphins are the major post-translational products of the prodynorphin (*Pdyn*) gene. They are opioid peptides that are derived from the prepro-Dyn precursor, along with enkephalins and endorphins, and contain the leucine (leu)-enkephalin sequence at the N-terminal portion of the molecule (155, 162, 163). There are two forms of Dyn (DynA and DynB). With the exception of DynA(2-13) (an inactive metabolite of DynA (1–17)), Dyns bind to all three opioid receptors (155). Dynorphins, especially DynA, have greater preference for KORs (164). Orexin promotes arousal (165) and is involved in modulating the rewarding effects of food (166, 167), sexual behavior (168), and drugs of abuse (55, 61). In contrast, Dyn facilitates depressive-like behavior and mediates the aversive effects of stress (33, 169). κ Opioid receptor activation has been shown to reduce the rewarding effects of drugs of abuse (156, 158) through actions that are at least partially mediated by midbrain DA systems (170, 171). Cocaine was shown to enhance the Dyn expression, KOR signaling, and KOR levels in both the VTA and striatum (172–176). Cocaine was also shown to upregulate *Pdyn* gene expression in the NAC (177). A decrease in KOR expression was found in the septum and BLA in animals that were withdrawn from binge COC self-administration (178). Despite the divergent influences on motivation, these two peptides closely interact. Orexin neurons in the LH express Dyn (32), and Orx and Dyn have been shown to act synergistically. Electrical stimulation of the HYP releases both Orx and Dyn (179). Orexin and Dyn are also co-released and play opposing roles in COC self-administration, brain stimulation reward, and impulsivity (180, 181). They also exert opposing effects in the VTA (181, 182), such as opposing actions on VTA neuronal firing rate, in which they counteract each other's effects upon co-release (181). OrxR1 antagonism in the VTA markedly reduced COC intake under a fixed-ratio 5 schedule, and this effect was blocked by KOR antagonism, suggesting that unopposed actions of Dyn within this brain region control COC intake (Figure 3; 181). The co-application of both Dyn and Orx caused no net changes in VTA DA neuron firing, suggesting that they exert balanced and opposing actions on DA neurons in the VTA (181)). Dynorphin has also been shown to attenuate the greater firing rate that is elicited by Orx in hypothalamic neurons (179) and counterbalance the response of basal forebrain neurons to Orx, preventing overexcitation (184).

## Cellular and behavioral evidence of opposing roles for dynorphin and orexin in the paraventricular nucleus of the thalamus

KOR mRNA expression is high in the PVT, where a high degree of correspondence between KOR mRNA and KOR binding was observed (185). Data from our laboratory suggest that Orx and Dyn have opposing effects on excitatory transmission in PVT neurons and OrxA-induced COC-seeking behavior. DynA decreased and OrxA increased PVT glutamatergic (GLUergic) transmission (183). The co-application of DynA and OrxA also counteracted each other's actions on synaptic activity. Behavioral data showed that the priming effect of OrxA was prevented by DynA only in animals that had a history of COC dependence (Figure 3). The effect of DynA was prevented by the KOR antagonist nor-binaltorphimine dihydrochloride, confirming that KORs mediated the DynA-induced blockade of OrxA's priming effect and supporting the hypothesis that KORs may be a target for treating disorders that are associated with greater Orx activity.

Orexin neurons have dense projections to the PVT, and the PVT network is mostly GLUergic. We found an increase in Orx-induced GLU release in PVT neurons, and this effect was comparable to previous findings in the HYP (186, 187), laterodorsal tegmental area (188), nucleus tractus solitarius (189), and neocortex (190). We observed a decrease in GLU transmission that was induced by DynA application, which was similar to effects that were reported in other brain regions (191, 192). Glutamate signaling is modulated by OrxA and DynA in opposite directions. The effects of OrxA were completely reversed by DynA. These two systems are suggested to balance each other in the regulation of PVT neuronal activity (183). Glutamate release in the PVT appears to be regulated by the Orx system, whereas DynA acts locally on presynaptic terminals. DynA and OrxA appear to exert their actions at different loci in the PVT through different neural circuits. Such differential neurocircuitry in the VTA was recently proposed, in which the sensitivity to OrxA was circuit-specific (182). The effects of PVT manipulations with regard to cellular actions were consistent with behavioral data, suggesting that there is a functional interaction between the Orx and Dyn systems.

Our recent study (183) found that intra-PVT injections of OrxA induced COC- and SCM-seeking behavior, and a greater effect was seen in COC animals (Figure 3). The observation that local injections of OrxA in the PVT induced COC- and SCM-seeking behavior strongly supports the hypothesis that Orx projections to the PVT are important in the modulation of reward-seeking behavior in general. An explanation for the general reinstating effects of OrxA for both COC and SCM could involve the known involvement of the Orx system in arousal. Orexin is well known to regulate general arousal (133), and the anticipation of food reward activates Orx-containing neurons in the PVT (134). The medial prefrontal cortex is one target of Orx-activated PVT neurons (135, 136), suggesting that Orx inputs to the PVT facilitate cortical activation that is linked to general arousal (137), which could explain the non-specific effect of intra-PVT OrxA injections on both COC and SCM reinstatement. Furthermore, we found that Dyn did not block SCM seeking that was induced by Orx [Figure 3; (183)], suggesting that although Orx and Dyn might work in tandem, their functional (behavioral) interaction changes following COC dependence. After long access to COC, the Orx and Dyn systems may become “sensitized,” reflected by a greater inhibitory effect of DynA and a greater priming effect of OrxA. Orexin and Dyn system dysregulation that is induced by COC has been previously reported. Using an operant model of reward seeking (i.e., conditioned reinstatement), we showed that the Orx system was engaged to a greater extent by drugs of abuse than by SCM (19, 66, 127). Cocaine appears to produce neuroadaptations in circuits that control the motivation for natural rewards.

Although hypothalamic Orx neurons represent an exclusive source of OrxA to the PVT (54, 125), DynA is expressed in the PFC and CeA [i.e., other brain regions that are connected to the PVT; (125, 154, 193–195)], which may also modulate PVT OrxA transmission. The PFC is implicated in the executive control of drug seeking (18, 196) and has projections to the PVT (125). The PFC is also suggested to supply a considerable Dyn input to the PVT, thereby modulating the effects of Orx. To our knowledge, however, the modulation of COC-seeking behavior by Dyn that is derived from sources other than hypothalamic Orx neurons to the PVT requires further investigation.

The mechanism by which Orx and Dyn in the PVT influence COC seeking is not fully understood. One tentative explanation is that COC induces neuroadaptive changes in HYP Orx/Dyn and consequently alter the PVT OrxR/KOR balance. This could explain the preferential role of Orx and Dyn in COC-seeking behavior vs. SCM-seeking behavior. Hypothetically, synaptic changes at the level of the PVT itself (i.e., modifications of synaptic strength, alterations of peptidergic signaling) or genomic and post-translational modifications may occur, thus affecting Orx and Dyn signaling. This will require further investigation.

## Conclusion

The mesocorticolimbic system, especially the VTA, is the focus of most research that implicates Orx in addiction, thus neglecting other systems (34, 55, 61, 62, 181, 197). The PVT receives Orx projections. The PVT is a key component of COC-seeking circuitry, and Orx transmission in the PVT mediates COC-seeking behavior via OrxR2 (127). Dynorphin is co-released with Orx. Our data show that such an interaction is not anatomically specific (i.e., not only in the VTA)—it also occurs in the PVT. Orexin and Dyn have opposing effects both on excitatory transmission in the PVT and behaviorally (183). Further characterization of the involvement of PVT Orx/Dyn transmission in compulsive drug seeking and the way in which the equilibrium between these co-released peptides is affected by drug experience is critically important for medication development for psychiatric disorders, such as drug addiction.

## Author contributions

All authors listed have made a substantial, direct and intellectual contribution to the work, and approved it for publication.

### Conflict of interest statement

The authors declare that the research was conducted in the absence of any commercial or financial relationships that could be construed as a potential conflict of interest.
